# Syndemics at play: chronic kidney disease, diabetes and COVID-19 in Pakistan

**DOI:** 10.1080/07853890.2021.1910335

**Published:** 2021-04-07

**Authors:** Inayat Ali

**Affiliations:** Department of Social and Cultural Anthropology, University of Vienna, Vienna, Austria

**Keywords:** COVID-19, pandemic, syndemics, CKD, diabetes

## Abstract

Although coronavirus disease 2019 (COVID-19) is a pandemic, it has several specificities influencing its outcomes due to the entwinement of several factors, which anthropologists have called "syndemics". Drawing upon Singer and Clair’s syndemics model, I focus on synergistic interaction among chronic kidney disease (CKD), diabetes, and COVID-19 in Pakistan. I argue that over 36 million people in Pakistan are standing at a higher risk of contracting COVID-19, developing severe complications, and losing their lives. These two diseases, but several other socio-cultural, economic, and political factors contributing to structured vulnerabilities, would function as confounders. To deal with the critical effects of these syndemics the government needs appropriate policies and their implementation during the pandemic and post-pandemic. To eliminate or at least minimize various vulnerabilities, Pakistan needs drastic changes, especially to overcome (formal) illiteracy, unemployment, poverty, gender difference, and rural and urban difference.

## Background

Undoubtedly, coronavirus disease 2019 (COVID-19) is a pandemic. Yet, it has several specificities due to the entwinement of the local historical, socio-cultural, environmental, economic, political factors, and structured vulnerabilities, which has caused a difference in the spread and effects of the virus in different countries [1–4]. Medical anthropologists have called this entwinement "syndemics" [5]. Currently, Bulled and Singer state, “COVID-19 syndemics in South Africa are likely to cluster among the economically, politically, and socially marginalized living in densely populated urban areas, where physical distancing and hygiene recommendations are nearly impossible to follow” [3]. Likewise, Pakistan has a vulnerable profile related to sociocultural, political, economic and healthcare system that creates a fertile ground for syndemics in which a communicable disease like COVID-19 to cause severe effects [6]. Like many low-income countries, Pakistan faces the prevalence of many communicable and non-communicable diseases that weaken the immune system while making a biosocial interaction and creating vulnerability to be infected by COVID-19 severely. That means at play are syndemics, which are critical biosocial health events involving a harmful interaction of a few diseases considerably shaped by socio-cultural patterns, environmental conditions, economic factors, and political systems [1]. Studies have revealed how various comorbidities have affected the outcome of COVID-19 worldwide [7–9]. In this article, I focus on COVID-19, chronic kidney diseases (CKDs), and diabetes in Pakistan to reveal syndemics at play.

## An overview of COVID-19 in Pakistan: preparedness and response

Pakistan reported the first infection of COVID-19 in two men who returned from Iran on 26 February 2020. By the first week of November 2020, the virus has infected and affected approximately 346,000 people, out of which around 7,000 have died [10]. Analogous to other countries, Pakistan has severely been affected by the pandemic, and specific socio-cultural, economic, and political factors add specificities to it [6,11]. As predicted, the “infodemic” and second wave are already overwhelming the country like the world demonstrating several underlying factors [12]. Some people have considered the entire COVID-19 outbreak as a “political game”, and yet others believe it is a “Western” and Jew’s “plot” [11,13–15]. Various and distinct rumours have circulated regarding the existence of COVID-19 and its potential treatment, such as drinking green tea [11–13].

To contain COVID-19, “flatten the curve”, and safeguard public health safety, the Pakistani government has taken several measures, which include suspending national and international travel, importing testing kits, opening quarantine centres, banning congregations, implementing lockdown, employing security forces, invoking Section 188 of the Pakistan Penal Code for violations of the ban, distributing food items among daily wage labourers, passing PKR1.2 trillion economic relief package, introducing “smart” lockdown to place virus hotspots under lockdown [6,10]. These steps seem promising, but if they bring a significant difference. The government seems engaged in playing several “political moves” to turn laypeople’s attention away from the drawbacks [16].

## Chronic kidney disease (CKD) and diabetes in Pakistan

### CKD

CKD is a serious health issue in high-income as well as low-income countries. It is an immediate or continual decrease in kidney function or efficiency for three months [17]. The epidemiological data reveal a significant increase in CKD, which is becoming an emerging global challenge [18]. At the scale of the Global Burden of Disease (GBD), CKD moved from the 27th position in the 1990s to the 18th in 2010 for significantly causing global deaths [19]. According to the same scale, its mortality rate has increased by over 134% as compared to 1990 [18]. The prevalence of CKD is higher while causing more deaths in some countries, especially in low-income countries [20]. Of approximately 500 million people having CKD worldwide, around 80% of them live in low- and middle-income countries (LMICs) [20]. Not only this, in China CKD and diabetic kidney disease (DKD) has significantly increased as compared to the past [21]. CKD is rapidly growing in Asian countries than in the global north. And, epidemiological transition in South Asian countries increases the risk factors of CKD [22–24]. There are several factors at play: behavioural, socioeconomic, and urbanization [18,21,22], which better be called syndemics.

In Pakistan, for instance, growing urbanizing exposes around 180 million people to chronic diseases, such as diabetes and hypertension, by the low birth weight associated with reduced renal reserve [22]. Also, diabetes and hypertension significantly contribute to CKD or end-stage kidney disease [18,21,22]. Specifically, in low-income countries, the significant reasons for CKD include diabetes mellitus (DM), hypertension (HTN), obesity, and cardiovascular disease [25,26]. This reveals an entanglement of numerous biosocial factors that make an individual prone to CKD. In March 2020, Pakistan’s *Dawn* newspaper reported that over 17 million people are suffering from kidney diseases in Pakistan that puts this country at the eighth number due to kidney diseases worldwide [27]. And, CKD is rapidly increasing due to kidney stone disease, increasing diabetes, and high blood pressure.

Due to reporting issues, it is difficult to estimate the number of people suffering from end-stage renal disease and needing renal replacement therapy (RRT) [28]. Although kidney transplantation is the most viable long-term option, transplant activities are seriously lagging behind demand, mainly due to insufficient financial support and a lack of organized transplant programs for deceased donors. Most implants come from living donors. Inadequate organ procurement networks, lack of facilities to supply potential donors, and inadequate public education result in the exploitation of deceased as well as living donors that led the Pakistani government to pass a law in 2007 and 2009 [28].

### Diabetes in Pakistan

Diabetes, a chronic disease, occurs when the pancreas is no longer able to make insulin or when the body cannot make fair use of the insulin it produces [29]. Without defining its three types—type 1 diabetes, type 2 diabetes, and gestational diabetes—I move forward to the epidemiological data, demonstrating that diabetes analogs to CKD, is a rapidly growing health challenge of the current century. According to the International Diabetes Federation [29], in 2000, 151 million adults lived with diabetes, which grew by 88% to 285 million in 2009. Currently, around 9.3% of adults aged 20–79 years that can be around 463 million, are living with diabetes (ibid.). Additionally, around 1.1 million children and adolescents under the age of 20 live with type 1 diabetes. An estimation was made in 2010 that there will be 438 million people with diabetes worldwide in 2025, but now the projection seems there will be around 25 million in 2025. Of these figures, as many as 19.4 million people live in Pakistan [30].

Similar to CKD, a rapid escalation of diabetes demonstrates a complex interplay of socio-cultural, economic, demographic, political, environmental, and genetic factors around the globe [29]. The risk factors for type 2 diabetes include expanding obesity, unhealthy diets, and widespread physical inactivity. The confounders are growing urbanization, changing lifestyle habits, such as higher calorie intake, increasing consumption of processed foods, and sedentary lifestyles (ibid.).

## Syndemics of CKD, diabetes and COVID-19

Several studies have highlighted the critical relationship between COVID-19 and existing comorbidities, including CKD and diabetes (see Table 1). Owing to the article's focus, the following sections discuss (a) syndemics of CKD and COVID-19; and (b) syndemics of diabetes and COVID-19.

**Table 1.** Characteristics of selected studies showing syndemics at play.

Authors	Study type	Publication year	Total no. of Patients	Comorbidities	Severe events	Definition of serious events
Chen et al. [32]	Retrospective	2020	274	Chronic hypertension and other cardiovascular comorbidities	113	Death
Cheng et al. [33]	Prospective cohort study	2020	701	Chronic kidney disease, chronic obstructive pulmonary disease, hypertension, diabetes, and tumour	294.4	Severely ill and death
Atkins et al. [34]	Cohort	2020	269,070	Hypertension, history of fall or fragility fractures, coronary heart disease, type 2 diabetes, and asthma	507	Critically ill and death
Barron et al. [41]	Whole population study	2020	61,414,470	Diabetes 1, diabetes 2 and other types of diabetes	7,869	Death
Onder et al. [43]	–	2020	355	Heart disease, diabetes, cancer, atrial fibrillation dementia, history of stroke	355	Death
Nasir et al. [46]	Observational study	2020	147	COVID‐19‐associated pulmonary aspergillosis, diabetes, and acute respiratory distress syndrome (ARDS)	23	ICU and death
Asghar et al. [47]	Single-center retrospective	2020	100	Diabetes, hypertension, ischaemic heart disease, CKD, and chronic liver disease	33	ICU and death
Zeb et al. [48]	Retrospective	2020	25	Hypertension, diabetes, CKD, ischaemic heart disease (IHD), chronic liver disease CLD and COPD	25	Death
Nandy et al. [39]	Systematic review and meta-analysis	2020	3,994	Hypertension (HTN), Diabetes mellitus (DM), Cardiovascular diseases (CVD), COPD and (CKD).	526	ICU admission, novel coronavirus pneumonia (NCP), mechanical ventilation, ARDS, and death
Emami et al. [7]	Systematic review and meta-analysis	2020	76,993	Hypertension, cardiovascular diseases, diabetes mellitus, smoking, COPD, malignancy, and CKD	3,403	Hospitalized patients
Yin et al. [37]	Systematic review and meta-analysis	2021	12,000	COPD, cardio-cerebrovascular diseases, diabetes, hypertension, and CKD	–	–
Singh et al. [35]	Systematic review and meta-analysis	2020	14 558	Hypertension, diabetes, CVD, COPD, CKD and cancer	–	Critical infection and death
Dorjee et al. [36]	Systematic review and meta-analysis	2020	38,906	Heart disease, COPD, CKD, CLD, hypertension, smoking history, diabetes	1,867.4	Severe disease and death
Guan et al. [8]	A nationwide analysis	2020	1,590	Hypertension, cardiovascular diseases, cerebrovascular diseases, diabetes, hepatitis B infections, COPD, CKD, malignancy and immunodeficiency.	254.4	Severe disease
Richardson et al. [9]	–	2020	5,700	Hypertension, obesity, and diabetes	553	Hospitalization, and Mortality

### CKD and COVID-19

The interaction of CKD and COVID-19 has been highly documented, resulting in severe complications, including deaths. Ssentongo et al. show that people underlying several diseases such as cardiovascular disease, hypertension, diabetes, and CKD, were at a considerable risk of mortality after being infected with COVID-19 as compared to those people who do not have these comorbidities [31]. In their sample of around 800 people, Chen et al. [32] found that 28% of those who died due to COVID-19 had CKD. Another study from China found 42% of 701 people infected with COVID-19 had comorbidities, in which CKD appeared decisive risk factor [33]. In England, CKD, and diabetes were among the significant pre-existing comorbidities that increase the risks to be infected by COVID-19 and develop critical complications, including deaths [34]. Three other studies showed a substantial relationship between CKD and critical COVID-19 complications [9,35,36]. In contrast, Yin et al. in 2021 found that although people with chronic obstructive pulmonary disease (COPD) and CKD seem at a low risk to contract COVID-19, they face critical effects once they are infected [37].

### Diabetes and COVID-19

Merril Singer has already revisited the interactive nature of diabetes and COVID-19 [1]. Several characteristics make diabetic people prone to COVID-19. For instance, it is said that the virus causing COVID-19 activates higher stress levels that then appears to greater release of hyperglycaemic hormones causing increased blood glucose levels [38].

Nandy et al. found in their systematic review and meta-analysis that diabetes mellitus (DM) have shown a substantial effect on the mortality rate in COVID 19 patients [39].

One study of around 45,000 COVID-19 infected people in China reported an overall case-fatality rate (CFR) of 2.3%, which was higher among people with underlying health conditions, such as 10.5% for people with cardiovascular disease and 7.3% for diabetes [40]. Similarly, another study in China with around 1600 infected people showed that people had diabetes developed critical COVID-19 infection [40]. In England, Barron et al. claim to cover almost England’s entire population and nearly the whole population diagnosed with type 1 and type 2 diabetes and found both types had greater odds of in-hospital death with COVID-19 than people without a diagnosis of diabetes [41]. In the USA, Muniyappa and Gubbi show that individuals with DM, hypertension, and severe obesity are at a significant risk to be infected and develop severe complications, including death, from COVID-19 [42]. In Italy, Onder et al. studied 355 people who died due to COVID-19, and of that, 35% (126) had diabetes [43]. Likewise, Huang et al. found that diabetes was significantly associated with severity, mortality, and acute respiratory distress syndrome in COVID-19 [44]. In India, Hussain et al. demonstrates that people with diabetes were at a higher risk of mortality and ICU admission [45]. In Pakistan, diabetes was among the considerable comorbidities in people infected with COVID-19 in Karachi and Rawalpindi [46–48]. 

## Syndemics of diabetes, CKD, and COVID-19 make Pakistan vulnerable

There are several intertwined reasons for interaction that explain the relationship between diabetes, CKD, and COVID-19 [1,38], which I do not go into details. The literature already cited shows that people with certain pre-existing comorbidities such as CKD and diabetes are at a higher risk of COVID-19 to be severely affected, including to die. Pal and Bhadada call the co-presence of diabetes and COVID-19 “an unholy situation” because both complement each other [49].

Nevertheless, based on the body of literature, one can easily add CKD into this “unholy” relationship since these prior comorbidities together multiply the severe effects. Owing to this “unholy” relationship, around 36.5 million people in Pakistan stay at a higher risk during this COVID-19 to develop a severe infection, including deaths.

## Conclusion

Syndemics of CKD, diabetes, and COVID-19 have been well studied now, globally. The evidence make a low-income country like Pakistan highly vulnerable in the face of a critical outbreak of COVID-19. Not only CKD and diabetes that have affected over 36 million Pakistan allow the novel coronavirus to significantly affect the country but also Pakistan’s institutionalized forms of socio-cultural, economic, and political disparities add further risks to the already at-risk populations. Like Mexico, as noted earlier, there are high rates of several communicable and non-communicable diseases, historical and structural factors in Pakistan. These syndemics—the biosocial [1] interactions—have roots in the country’s history of national and global governance that have encouraged Pakistan’s dominant class to structure specific webs resulting in many critical states such as increasing poverty, corruption, dependency on foreign aid, malnutrition, critical environmental effects of anthropogenic climate change. Around 25% of Pakistan’s population still lives below the poverty line; a significant number of women and children are malnourished, corruption is chronic and growing; climate changes are causing floods, earthquakes, heatwaves, and fog in the country; and there is a dearth of sufficient and efficient healthcare services and providers.

## Practical suggestions

Both the pandemic and syndemics have reminded a country like Pakistan to devise and implement effective and appropriate policies to overcome these vulnerabilities that result in critical “biosocial events”. During the pandemic, the government needs appropriate policies and implementation to protect a substantial number of people with underlying conditions and be ready for dealing with critical consequences of the entwinement of syndemics. While, after the pandemic, the country needs rapid and substantial changes to increase the rate of formal education, enhance the healthcare services and provision, decrease the growing poverty, overcome the gender gap, reduce the difference between rural and urban areas, eliminate malnutrition, minimize climate changes, and deal appropriately with a rapidly growing population. Otherwise, the structural vulnerabilities will keep Pakistan always at the top allow communicable and noncommunicable diseases to affect thousands of people and keep them in the face of syndemics to substantially impact a considerable number of people.
